# Supplementary material for: Endoplasmic reticulum network heterogeneity guides diffusive transport and kinetics
Source: Biophys J. 2023 Jul 3;122(15):3191–205. doi: 10.1016/j.bpj.2023.06.022 (PMC10432226; doi:10.1016/j.bpj.2023.06.022)
Supplement: Document S1. Supporting material [file mmc1.pdf]

Biophysical Journal, Volume *122*

Supplemental information

Endoplasmic reticulum network heterogeneity guides diffusive transport and kinetics

Zubenelgenubi C. Scott, Katherine Koning, Molly Vanderwerp, Lorna Cohen, Laura M. Westrate, and Elena F. Koslover

S1. SPATIALLY HETEROGENEOUS ACCESSIBILITY OBSERVED FOR MULTIPLE ER STRUCTURES

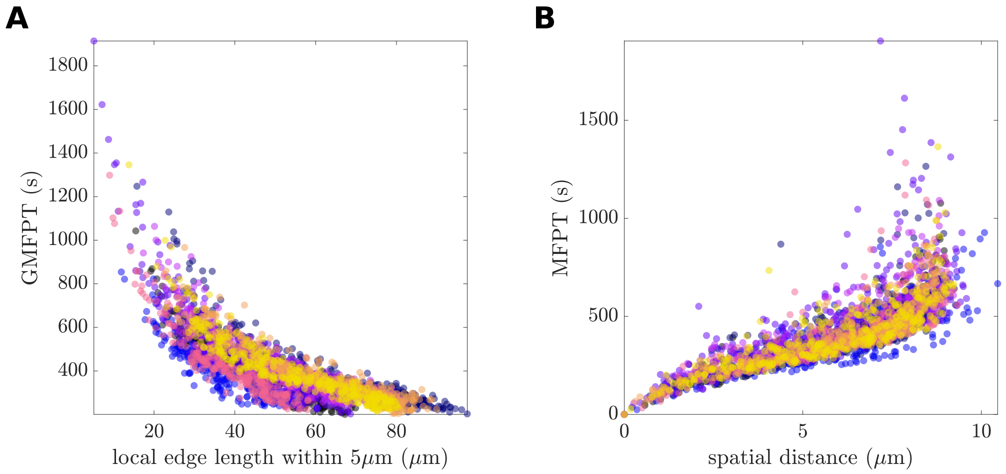

FIG. S1: Diffusive search times for extracted ER network structures. (A) Global mean first passage time (GMFPT) to each node in the network is plotted against the local network edge length (L_{loc}) within $\sigma = 5$ μm of the node. Compare to Fig. 1D. (B) Mean first passage time (MFPT) from a central node to target nodes on the ER network is plotted against Euclidean distance to the target node. Compare to Fig. 1F. In both plots, color indicates results for individual ER architectures (N=8 network regions extracted from 3 cells).

High spatial heterogeneity in network density and diffusive accessibility is observed across multiple excised ER networks. Using eight circular regions (radius 8.5 μm) of extracted ER network structure, local edge lengths and mean first passage times are calculated, as for the example network shown in Fig. 1.

The global mean first passage time (GMFPT) to every node in each network (Fig. S1A) again scales inversely with local edge length. In individual networks, GMFPT can vary by nearly one order of magnitude with similar variation observed in the distributions of local edge length. Notably, the variation within a single network structure is comparable to the overall variation between networks extracted from different cells. Thus, even a well-connected, highly-looped structure like the ER can exhibit large spatial fluctuations in diffusive accessibility, an effect that is observed across multiple peripheral ER architectures.

We next consider a more localized search process wherein a particle begins its search from a central source node (Fig. S1B). The mean first passage time (MFPT) to each possible target node in the ER structures exhibits the characteristic scaling with distance previously discussed in the main text and elsewhere [1]. Fluctuations in ER density and connectivity lead to large deviations from the smooth scaling seen in uniform networks (see Fig. 1E). This implies that in the ER search times for targets that are equidistant from a central source can vary substantially depending on the local geometry.

S2. COMPUTING MEAN FIRST PASSAGE TIMES ON RESERVOIR NETWORKS

Our base model treats regions of the ER as networks of edges connected at point-like junctions. This model assumes that the size of the network junctions is comparable to the tubule width. However, the peripheral ER can also include some sheet-like regions that are substantially larger

than the tubule width [2]. Such peripheral sheet regions would form spatially expanded junctions that could serve as localized traps for diffusing particles. We therefore consider an expansion of our model that incorporates enlarged junction regions from which particles escape through narrow holes along the boundary.

In addition, the perinuclear region of the ER forms a complex structure composed of interconnected stacks of sheets. The detailed structure of the perinuclear ER cannot be resolved with confocal microscopy, but given the large extent of the region we might also expect it to serve as a substantial trap for diffusing particles. A simplified representation of the perinuclear region would also treat it as a disk-shaped reservoir connected to many tubules around its circumference. We use the same mathematical approach to handle the large perinuclear region as we do for the enlarged junction nodes. This approach is described below.

We propose a "reservoir network" model for calculating analytic mean first passage times in a structure composed of tubes that are joined either at point-like nodes or at larger reservoirs. The approximate analytical approach for transitions between reservoirs and tubes is inspired by previous work describing diffusive escape from pores in cylindrical geometries [3].

In essence, the model represents transitions of particles between disk-shaped reservoirs and connecting tubules (Fig. S2A) by decomposing the system into several discrete spatial states (Fig. S2B), in a manner analogous to the classic paper on diffusion-limited reactions by Berg and Purcell [4]. By using a state-based approach, it is then possible to calculate MFPTs on these hybrid reservoir networks using the previously described graph-theoretic approach [5].

In Supplemental Section S3 we apply this model to study the effect of scattered reservoirs on average search times on a network. Additionally, in the main text, a single reservoir representing the sheet-like ER in the perinuclear region is used to more accurately capture search times on a whole-cell scale.

S2A. State diagram for a single reservoir

Reservoirs are treated as disks of radius R_+, with holes in the boundary of width 2δ representing connected tubes (a typical geometry is shown in Fig. S2A with the corresponding state diagram in Fig. S2B). An optional target at the center with radius R_- is denoted the central state, C. When a particle enters the reservoir from an adjoining tube it is placed in the boundary state, B, a distance σ from the outer wall of the reservoir. The value of σ is chosen to be comparable to the tube size, and represents a distance from which the particle can take independent samples of the domain wall without maintaining a strong memory of the tubule from which it came. From state B, the particle can either transition back towards the wall or take the long journey to the center of the reservoir, with splitting probabilities ($\mathcal{E}_\pm$) and average waiting time until the transition (Q_B) given by:

$$\begin{aligned}
\mathcal{E}_+ &= \log\left(\frac{R_+ - \sigma}{R_-}\right) / \log\left(\frac{R_+}{R_-}\right), \\
\mathcal{E}_- &= 1 - \mathcal{E}_+, \\
Q_B &= \frac{1}{4D\log\left(\frac{R_-}{R_+}\right)}\left((R_+ - \sigma)^2 \log\left(\frac{R_+}{R_-}\right) + R_+^2 \log\left(\frac{R_-}{R_+ - \sigma}\right) + R_-^2 \log\left(\frac{R_+ - \sigma}{R_+}\right)\right).
\end{aligned} \tag{S1}$$

Here D is the particle diffusivity, $\mathcal{E}_+$ is the probability of hitting the wall before the central target, and $\mathcal{E}_-$ is the probability of first hitting the central target. If there is no target at the center of the reservoir, then $R_- \to 0$ and $\mathcal{E}_+ = 1$. These expressions are derived from the standard solution of first passage times for the diffusion equation in cylindrical coordinates [6].

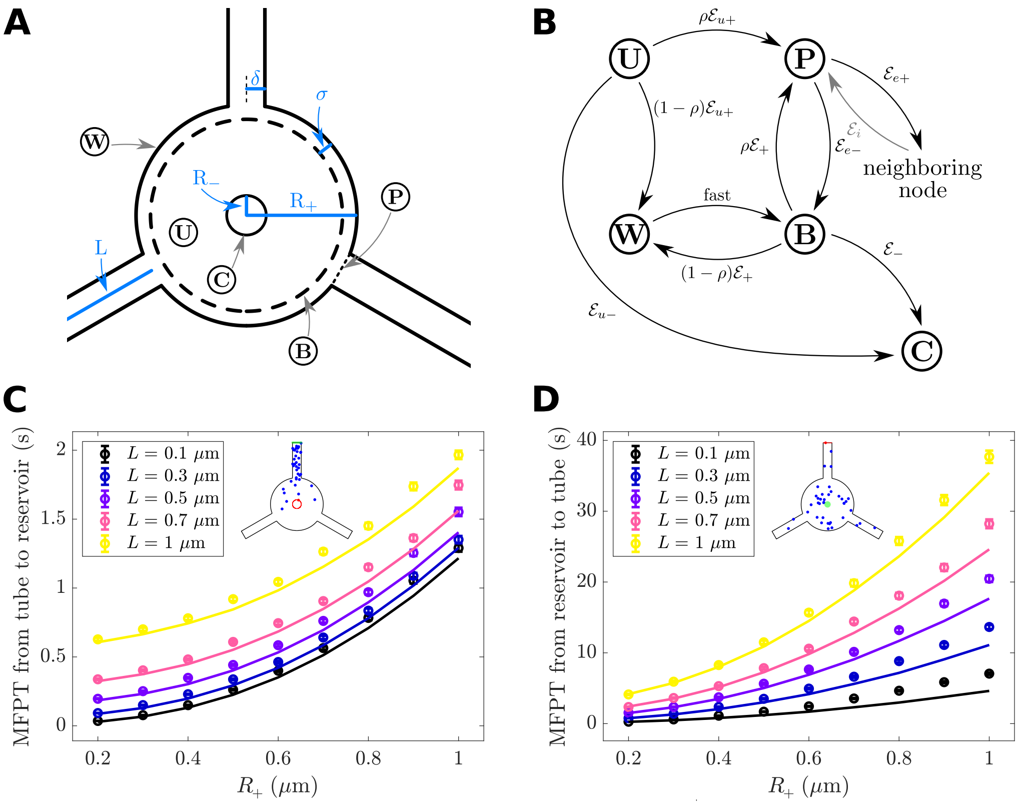

FIG. S2: Reservoir network model. (A) Representative geometry for a three-tube reservoir embedded in the network. Dimensions of the system are highlighted in light blue. The five states and their locations in space are denoted by circled capital letters. (B) The state diagram for the effective model with transition probabilities labeled. The reservoir may be embedded in a larger network, so an additional neighboring node state is depicted where the transition into P from the neighbor is given as in [5]. (C) Analytic MFPTs (lines) reproduce MFPT from BD simulations (points) of 2000 particles diffusing ($D = 1\ \mu\text{m}^2/\text{s}$) across wide range of symmetric triskelion geometries. Error bars show standard error of the mean. Inset: particles (active, blue; absorbed, red) start at the green tip of one tube and diffuse until reaching the red target at the center of the reservoir. (D) Simulations validate the reverse process. Inset: particles start at the center of the reservoir (green) and diffuse until reaching the tip of the top tube (red).

If the particle first hits the wall, it has a chance $\rho = \frac{\delta d}{\pi R_+}$ of entering a P state, where d is the number of tubules connected to the reservoir. The particle is equally likely to hit each P state (this does not mean it will reach the connected node with equal probability, which depends on the length of the intervening edge). The P state can be thought of as a network node connected to two edges: one of length L leading to the neighboring node outside the reservoir and one of length σ leading into the reservoir. The splitting probability from P to B is simply $\mathcal{E}_{e-} = \sigma/(L+\sigma)$. If the particle instead hits the wall, W (probability $1-\rho$), it rapidly transitions back to state B. Assuming σ is small compared to R_+, the waiting time in W will be negligible compared to the time spent performing searches of the bulk. This approach for exit out of a reservoir through a long tube has been previously described for cylindrical geometries [3].

Included in the state diagram is an additional state, U, an optional initial state representing a uniform distribution of particles throughout the reservoir. The splitting probabilities and waiting

times unique to state U are given by:

$$\begin{aligned}
\mathcal{E}_{u+} &= \frac{R_+^2}{R_+^2 - R_-^2} + \frac{1}{2\log\left(\frac{R_-}{R_+}\right)}, \\
\mathcal{E}_{u-} &= 1 - \mathcal{E}_{u+}, \\
Q_U &= \frac{1}{8D}\left(R_-^2 + R_+^2 + \frac{R_-^2 - R_+^2}{\log\left(\frac{R_+}{R_-}\right)}\right).
\end{aligned} \tag{S2}$$

For simplicity, this state diagram represents a reservoir with one connected tubule. In practice, there are often multiple P states, each connected to a neighboring node. Then the transitions from U and B to each P_i would be symmetric and scaled by the degree (number of neighbors) of the reservoir.

This reservoir model can be inserted into previously described network models of the ER. Using the splitting probabilities and waiting times for each state, it is possible to calculate analytic mean first passage times for diffusive search in the reservoir as previously described in [5].

S2B. BD simulations in triskelion geometries validate analytic approach

The analytic model is validated through the use of Brownian dynamics simulations on triskelion geometries with a wide range of sizes (Fig. S2C,D insets). The radius of the reservoir, R_+ is varied between $0.2 - 1.0$ μm and the length of the connected tubules is varied from $0.1 - 1.0$ μm. The radius of the tubes is fixed at $\delta = 0.05$ μm, a typical size for ER tubules in COS7 cells [2, 7]. The modified tube size, is set to $\sigma = \pi\delta/4$ as in [3, 4]. In all of these tests (and later applications), the central target has radius $R_- = \delta$ so that targets placed in either a tube or a reservoir have the same size.

Two representative search problems are analyzed on these geometries. In the first test, 2000 particles are placed at the end of a tube and allowed to diffuse until reaching an absorbing disk at the center of the reservoir (Fig. S2C). In the second, 2000 particles begin at the center of the reservoir and diffuse until reaching the end of a specific tube. The time step for both tests is 10^{-5} s, with particle diffusivity $D = 1$ μm^2/s. The MFPT is recorded and compared to the analytic MFPT from the corresponding reservoir network (Fig. S2C,D).

Over a wide range of scales for both R_+ and L the analytic model approximately reproduces simulated mean first passage times. Having validated a single triskelion, it is now possible to intersperse these reservoir structures throughout existing networks and obtain realistic mean first passage times on reservoir networks.

S3. PRESENCE OF SCATTERED RESERVOIRS INCREASES TRAPPING, GMFPT

By treating the peripheral ER as a network of one-dimensional tubules we are able to focus on large-scale structure and connectivity. This enables us to model transport in the ER as diffusion on a network, where simple computational approaches provide powerful predictions that agree with experimental results (Fig. 2E). Here we investigate the limits of the network model by considering the effect of scattering enlarged junction reservoirs over a network.

A COS7 peripheral ER is modeled as a reservoir network by randomly selecting 20% of its nodes and converting them into reservoirs. The reservoir radii (R_+) are set to be 0.45 of the minimum

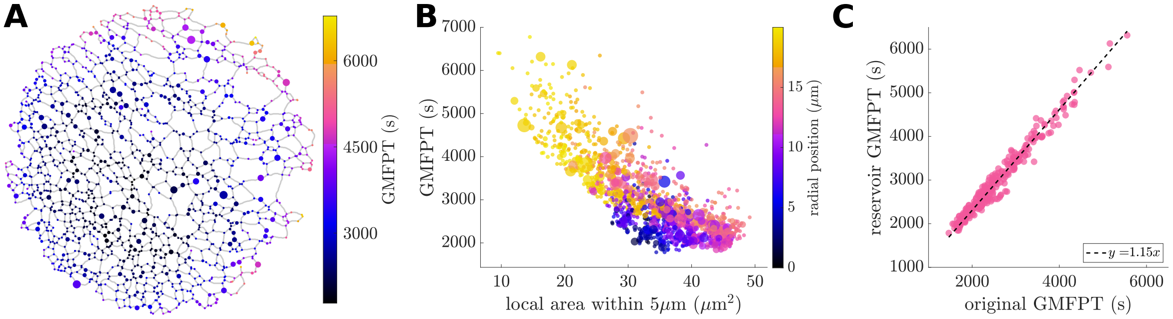

FIG. S3: Reservoir network model of a COS7 ER. (A) Global mean first passage times (GMFPTs) to targets on nodes on an example COS7 ER network (from Fig. 1A) where now 20% of network nodes are converted to reservoirs. Reservoir nodes are shown as true size, point-like nodes are plotted with a fixed minimum size for visibility. (B) GMFPT scales inversely with local network surface area. Color denotes radial position from center of network, enlarged points indicate reservoirs and their relative size. (C) The GMFPT to central targets in reservoirs scales linearly with GMFPT of the original nodes (prior to conversion to reservoir).

edge length connected to the node, giving an average size of 0.26 μm. Connected edges shrink to accommodate the reservoirs. This avoids unphysical overlap of reservoirs and edges and allows for a heterogeneous distribution of reservoirs to be randomly dispersed across the network. The GMFPT is calculated to targets of radius $R_- = 0.05$ μm placed in the center of each expanded node, as well as to all the ordinary point-like junctions. Because the particles are meant to represent ER membrane proteins, the starting probability for each node is set in proportion to surface area, with non-reservoir nodes assigned surface area $2\pi\delta^2$, while reservoirs have surface area $2\pi R_+^2$.

We find that even in the presence of many reservoirs, the qualitative trends for network search times are the same. GMFPT still scales with the amount of locally accessible network (in this case, surface area, Fig. S3B). For each individual reservoir target, we compare its GMFPT in a network with many reservoirs versus the GMFPT to the same node on a network with point-like junctions only (Fig. S3C). The main effect of enlarged junction reservoirs is a slight increase in search times. This is due to the increase in network surface area and the trapping that occurs inside of reservoirs; there is more space for particles to explore before finding any given target and thus average search times increase. Notably, the breadth of the search-time distribution does not change substantially upon the introduction of randomly scattered enlarged reservoirs. Thus, treating the ER tubule junctions as point-like connections appears to be a reasonable simplification for these network structures.

S4. ALTERNATIVE METHODS OF INCORPORATING NETWORK DYNAMICS INTO SIMULATIONS OF PARTICLE DIFFUSION

When modeling the spread of photoactivated diffusing particles from a central region, the dynamic rearrangements of the ER network need to be considered. In the main text, we incorporate the effects of the time-varying ER structure by averaging multiple simulations on static networks extracted from individual frames of the experimental movie. Here, we analyze two alternatives: (1) simulations run only on a single network extracted from the first frame; and (2) a "project and propagate" approach incorporating many network snapshots in each simulation. In both cases, the same two rounds of filtering are applied when comparing to the experimental signal.

For approach (1), the simulated fractional signal on the network extracted from frame 1 (f_{1ij}^{sim}) is plotted in Fig. S4A.ii. We calculate the slope of the signal in each wedge j over the first 10

seconds after photoactivation. These arrival rates are compared to the averaged simulated rates from the main text (computed from the averaged fractional signal shown in Fig. S4A.iii). We see strong agreement between the two ($R^2 = 0.91$, Fig. S4B, blue dots and dashed line). Consequently, this approach also does reasonably well in predicting the experimentally measured protein arrival rates (extracted from Fig. S4A.i), with $R^2 = 0.6$ (Fig, S4C, blue dots and dashed line). Approach 1 includes no information about the network rearrangements over time, but still captures much of the behavior of proteins in the ER.

For the project and propagate simulations, 50000 particles commence diffusion from the photoactivation region on the network extracted from the first frame of the movie. The photoactivated particles then diffuse along the first network for time $dt = 0.6$s, at which point they are projected from their location in space to the closest point on the network extracted from the second frame of the experimental movie. The projection step tends to be small compared to the diffusive (or propagation) step because the network evolves slowly in time compared to the diffusion of individual particles. The diffusive particles repeat this process, propagating and projecting, until the final frame of the experiment is reached.

One advantage of this method is that it more realistically captures the expected spatial trajectories of particles diffusing on a continuously rearranging network. As time progresses and the proteins diffuse from the photoactivation region, the network evolves in time as well, albeit with a large time step set by the experimental frame rate.

The same nine photoactivation runs are analyzed using the project and propagate simulation method. We again define individual wedge regions of the same size and location as in the experimental images and analyze the number of particles in each. The simulated signal in each wedge (w_{ij}^{pp}) is then defined as the total number of particles in wedge j at time point i, and the fractional signal (Fig. S4A.iv) is $f_{ij}^{\mathrm{pp}} = w_{ij}^{\mathrm{pp}}/N$. These fractional signals do not require averaging as in the main text, because the effects of the dynamic network are already incorporated via the project and propagate method. The fractional signals are then used to find the signal arrival rate (slope over first 10 seconds), just as before.

Again, we see strong agreement between the project and propagate simulations and the results from the main text when comparing the calculated arrival rates (Fig. S4B). The project and propagate method is also able to predict the experimental arrival rates well: $R^2 = 0.64$ (Fig. S4C), as compared to $R^2 = 0.68$ in the main text.

Both project and propagate and the frame-averaged approach outlined in the main text generate simulated protein arrival rates that fit the experimental rates slightly better than the static approach using the first frame only. These approaches for incorporating ER network dynamics thus help account for the time-varying tubule density and connectivity within each ER region. Thus, although ER network dynamics are shown to be quite slow, incorporation of the different network structures over time allows for a better representation of observed particle motion.

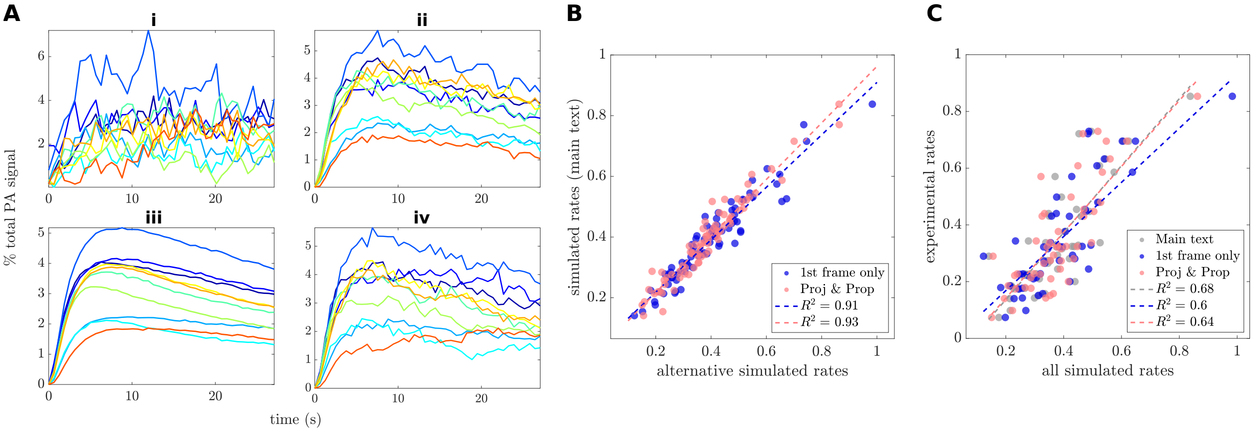

FIG. S4: Alternative simulations of diffusive spread in the ER. (A) Fractional photoactivated signal vs time curves for a single cell from experiment and simulations: (i) experimental signal, (ii) simulated signal on the network from the first frame, (iii) simulated signal averaged over networks from each frame of the experimental movie (as used in main text, Fig. 1E), and (iv) simulated signal from the project and propagate approach. (B) Correlation of signal arrival rates from alternative simulation approaches with the averaging approach used in the main text. (C) Correlations of simulated rates with experimental protein arrival rates.

[1] M. Dora and D. Holcman, Proceedings of the Royal Society B **287**, 20200493 (2020).

[2] L. K. Schroeder, A. E. Barentine, H. Merta, S. Schweighofer, Y. Zhang, D. Baddeley, J. Bewersdorf, and S. Bahmanyar, J Cell Biol **218**, 83 (2019).

[3] Z. Yang and E. F. Koslover, Phys Biol (2023), URL `http://iopscience.iop.org/article/10.1088/1478-3975/acb1ea`.

[4] H. C. Berg and E. M. Purcell, Biophysical journal **20**, 193 (1977).

[5] Z. C. Scott, A. I. Brown, S. S. Mogre, L. M. Westrate, and E. F. Koslover, Eur Phys J E **44**, 1 (2021).

[6] S. Redner, *A Guide to First-Passage Processes* (Cambridge University Press, 2001).

[7] T. Konno, P. Parutto, D. M. Bailey, V. Davì, C. Crapart, M. A. Awadelkareem, C. Hockings, A. Brown, K. M. Xiang, A. Agrawal, et al., bioRxiv (2021).
